# Multisource feedback analysis of pediatric outpatient teaching

**DOI:** 10.1186/1472-6920-13-145

**Published:** 2013-11-01

**Authors:** Mao-Meng Tiao, Li-Tung Huang, Ying-Hsien Huang, Kuo-Shu Tang, Chih-Jen Chen

**Affiliations:** 1 Department of Pediatrics, Kaohsiung Chang Gung Memorial Hospital, Chang Gung University College of Medicine, Kaohsiung, Taiwan

**Keywords:** 360 degree, Video-tape, Education, Outpatient

## Abstract

**Background:**

This study aims to evaluate the outpatient communication skills of medical students via multisource feedback, which may be useful to map future directions in improving physician-patient communication.

**Methods:**

Family respondents of patients, a nurse, a clinical teacher, and a research assistant evaluated video-recorded medical students’ interactions with outpatients by using multisource feedback questionnaires; students also assessed their own skills. The questionnaire was answered based on the video-recorded interactions between outpatients and the medical students.

**Results:**

A total of 60 family respondents of the 60 patients completed the questionnaires, 58 (96.7%) of them agreed with the video recording. Two reasons for reluctance were “personal privacy” issues and “simply disagree” with the video recording. The average satisfaction score of the 58 students was 85.1 points, indicating students’ performance was in the category between satisfied and very satisfied. The family respondents were most satisfied with the “teacher”s attitude,“ followed by ”teaching quality”. In contrast, the family respondents were least satisfied with “being open to questions”. Among the 6 assessment domains of communication skills, the students scored highest on “explaining” and lowest on “giving recommendations”. In the detailed assessment by family respondents, the students scored lowest on “asking about life/school burden”. In the multisource analysis, the nurses’ mean score was much higher and the students’ mean self-assessment score was lower than the average scores on all domains.

**Conclusion:**

The willingness and satisfaction of family respondents were high in this study. Students scored the lowest on giving recommendations to patients. Multisource feedback with video recording is useful in providing more accurate evaluation of students’ communication competence and in identifying the areas of communication that require enhancement.

## Background

Communication is an important component of patient care. The physician-patient interview is the key component of all health care, particularly of primary medical care [1,2]. Outpatient clinics offer trainees one of the most varied clinical experiences within the hospital setting, but they are often chaotic and over-stretched, with limited time for teaching [3]. When patients are informed and involved in decision making, they adhere to medical recommendations (e.g., vaccination and dietary modification) [1,4]. Such joint decision making requires patients to be fully informed about alternatives and potential risks of treatment [1,5]. Clinical practice within outpatient clinics can strengthen the collective knowledge of trainees [3]. This establishment of practice may also validate the role of trainees in the management of patients and facilitate social learning [3].

Assessment is an essential step in the curricular development process [6]. An evaluation method is important for the improvement of the quality of learning among medical students; however, such method is rare [6]. Concern about the inability of monitored examinations to assess the full spectrum of clinical competence, including humanistic quality, knowledge, and communication skills, stimulated the introduction of the “patient and peer assessment module” [7]. On the other hand, supervision features observation and sharing of clinical feedback, which can improve clinical performance [3]. Effective needs-assessment strategies include Multisource feedback (MSF) from educators and learners [8]. Thus, the patient and peer assessment, and self-evaluation modules are better methods to evaluate the communication and clinical performance of medical students [7].

We aimed to evaluate the outpatient communication skills of medical students by using MSF from family respondents of patients, nurses, a clinical teacher, and a research assistant. This may be an effective evaluation method in the future to improve physician-patient communication skills in the outpatient setting.

## Methods

### Settings: undergraduate students of Chang Gung Memorial Hospital (CGMH)

We employed a multi-respondent evaluation method using a structured paper questionnaire to investigate the communication skills of our students in the pediatric outpatient clinic. All seventh-year medical students trained at CGMH were enrolled in this study. There were 32 males and 28 females, with mean age of 25 years (24–27 y). MSF was obtained from a nurse, a clinical teacher, and a research assistant to assess the medical students’ communication competence; a self-assessment of skills was also administered by the students. The satisfaction score of the medical students was evaluated by 4 respondent groups which included the family, research assistant, nurse and students. The same teacher and the same research assistant were involved in the whole study. The patients, 36 males and 24 females, were one month to 16 years old (median: 3.3 y). The patients who were suitable for this teaching clinic were classified by nurses before the patients went into the outpatient clinic. The study was approved by the Ethics and Clinical Research Committee of CGMH (No. 98-2202B).

### Instruments

Each outpatient interaction led by the medical students was video-recorded. The students, a nurse, a teacher, and a research assistant, watched the video together in a room at the same time and then filled out the corresponding paper questionnaire. The observers (students, nurse, teacher, research assistant) discussed the videotaped interactions after completing the assessments. The family respondents completed reasons for agreeing/disagreeing to the questionnaires before video recording and completed their assessments after the outpatient clinic at a room next to the clinic.

### Assessment and evaluation

**Figure 1:**
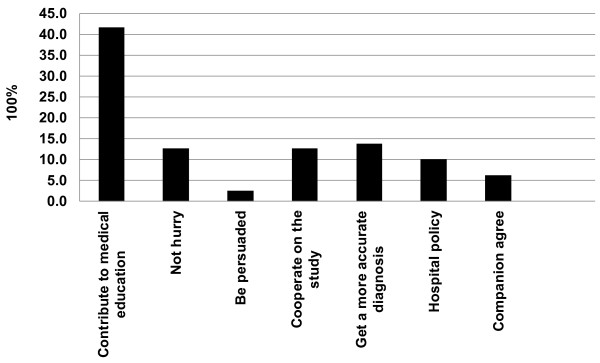
**Agree reasons.** Patients agreed with the video recording to contribute to medical education, to get a more accurate diagnosis, and to cooperate on the study.

**Figure 2:**
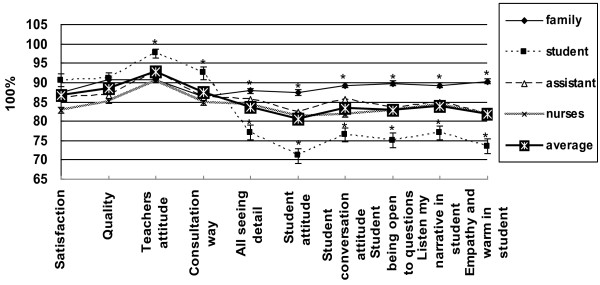
**Teaching satisfaction.** Average satisfaction score was 85.1 points. Students scored highest on “teacher’s attitude,” followed by ”teaching quality.” Family respondents were most satisfied with “teacher’s attitude” followed by “teaching quality”; students were most satisfied with “teacher’s attitude” and “being open to questions.” The family respondents of patients were least satisfied with “being open to questions,” whereas students were least satisfied with “student’s attitude.” The results represented were mean ± SD. * P < 0.05 compared to the average scores.

**Figure 3:**
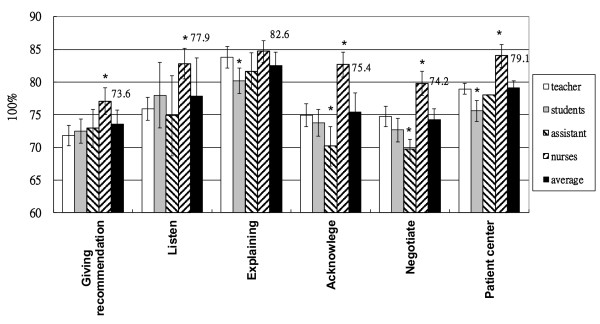
**Outpatient skills in six assessment domains.** Students scored highest on “explaining” and lowest on “giving recommendations” as in the average scores. The results represented were mean ± SD. * P < 0.05 compared to the average scores.

The completed questionnaires were validated by 3 professional teachers. The questionnaire included items such as family respondent’s reasons for agreeing/disagreeing with the video recording, multisource satisfaction with the outpatient interaction, and evaluation of skills in 6 domains (i.e., giving recommendations, listening, explaining, acknowledging, negotiation, and patient-centered communication). These skills were subdivided accordingly as shown in Table 1 and Figures 1, 2, 3[1,9]. Every question can be answered with the following options and corresponding scores: very satisfied (100 points), satisfied (80 points), no opinion (60 points), dissatisfied (40 points), and very dissatisfied (20 points).

**Table 1:** Subdivided outpatient skills evaluation by the different groups

		**Teacher mean (SD)**	**Students**	**Assistant**	**Nurses**	**Average**	**p value**
Giving recommendation	Clearly tell the patient examination and treatment plan can be selected	75.5 (14.5)	73.2 (14.1)	76.1 (11.4)	79.0 (14.9)	76.0 (14.7)	0.199
	Clear description of the procedures of the entire treatment plan	74.8 (13.3)	72.5 (15.9)	76.1 (12.0)	78.0 (16.8)	75.4 (15.4)	0.269
	Medical services to provide disease prevention or health promotion (vaccines, diet, exercise …)	66.6 (15.6)	70.2 (14.5)	71.5 (14.2)	75.3 (16.6)	70.9 (16.8)	0.040
	When should I seek further medical care or back to the outpatient	71.0 (14.6)	73.2 (15.8)	70.8 (14.9)	79.0 (16.2)	73.5 (14.3)	0.020
	Taking into account the holistic care of the physical, psychological and social	71.0 (12.5)	73.6 (14.7)	70.2 (11.9)	74.0 (16.6)	72.2 (14.9)	0.413
Listen	Patients understand the words to communicate	87.6 (10.5)	81.0 (12.4)	84.9 (9.4)	87.0 (12.1)	85.1 (12.6)	0.018
	**Asking about chief complaints**	81.0 (10.9)	99.7* (13.3)	93.4* (10.2)	88.0* (9.9)	90.5* (8.4)	0.673
	Know the patient’s entire medical history	80.3 (9.5)	79.0 (12.1)	81.0 (6.8)	87.7 (9.8)	82.0 (11.4)	0.001
	Encourage patients to express the suffering of all physical symptoms	79.3 (7.5)	80.0 (11.1)	80.0 (8.2)	85.7 (12.3)	81.2 (11.5)	0.007
	Deep ask the patient’s main problem	79.0 (8.7)	76.9 (14.1)	75.4 (12.3)	84.0 (12.6)	78.8 (13.4)	0.003
	Encourage patients to express their concerns about physical symptoms	71.4 (15.0)	74.6 (14.3)	69.8 (14.9)	81.7 (10.6)	74.4 (11.5)	0.001
	Deep understanding of the patient to the clinic	73.4 (13.7)	72.5 (14.1)	67.5 (6.4)	79.7 (10.4)	73.3 (11.2)	0.001
	Know the emotional needs of the patients	64.5 (15.0)	71.2 (12.8)	66.6 (7.4)	78.0 (7.9)	70.1 (11.3)	0.001
	Deep understanding of the patient to the clinic grounds	80.7 (11.2)	78.3 (12.7)	77.7 (7.7)	86.3 (11.3)	80.8 (13.4)	0.001
	**Ask the decision-makers and caregivers of the patient’s disease**	94.5* (9.8)	77.6 (13.2)	73.8 (10.0)	86.0 (10.6)	83.0 (14.4)	0.001
	**Asking about life/school burden**	59.7** (12.7)	66.4** (17.3)	62.3** (15.5)	74.0** (8.9)	65.6** (15.1)	0.001
	**Family or personal life effect**	**60.0 (14.0)**	78.3 (7.9)	65.6 (15.5)	75.7 (8.8)	69.9 (12.6)	0.066
Explaining	**Self-introduction**	**90.0 (18.1)**	83.1 (17.5)	83.6 (14.4)	85.7 (15.2)	**85.6 (17.3)**	0.120
	To do a complete description of the patient’s condition	77.6 (13.5)	77.3 (15.8)	79.7 (9.3)	83.7 (13.5)	79.6 (13.3)	0.055
Acknowledge	Encourage the patient to have questions about what had explained	79.0 (8.7)	76.3 (15.0)	74.4 (11.0)	86.0 (12.9)	78.9 (13.8)	0.001
	Allow patients to fully ask questions related to their symptoms	78.6 (8.3)	75.9 (14.7)	72.8 (7.7)	85.0 (12.6)	78.1 (14.2)	0.001
	**Get a consensus with patient in different views**	67.2 (13.9)	69.2 (16.4)	63.3 (16.4)	77.0 (15.5)	69.2 (12.6)	0.001
Negotiate	Encourage patients to present themselves for examination or treatment	76.2 (10.9)	71.2 (17.1)	69.8 (14.0)	78.3 (7.8)	73.9 (14.2)	0.010
	Ask the patient’s view about physician’s examination or treatment	72.4 (13.9)	71.2 (15.3)	71.1 (12.4)	80.0 (16.1)	73.7 (14.6)	0.003
	Discussed with the patient about the examination or treatment plan and get the patient's consent	75.5 (12.4)	75.6 (14.1)	68.2 (12.8)	81.0 (14.9)	75.1 (13.2)	0.001
Patient center	Patient center	79.0 (6.9)	75.6 (12.8)	78.0 (8.7)	83.0 (12.2)	78.9 (12.3)	0.010

* best performance, ** worst performance.

Our patients included children with respiratory tract infection, liver cirrhosis, and abdominal pain, and those requiring post-operative care. These patients were classified into difficult or common cases evaluated by students after the clinics. The liver cirrhosis patients included hepatitis or biliary atresia with routine follow-up and without complications.

The questionnaires in this study were tested with Cronbach’s α for reliability. Statistical methods employed were descriptive statistics and student’s t-test (one-sided) in comparison between difficult and common cases. Our hypothesis is that the student will have lower scores with difficult cases. Comparisons in teaching satisfaction and six domains between different respondent groups were analyzed by the ANOVA with Bonferroni’s correction, when multiple comparisons were evaluated. The correlation in the overall satisfaction with number of questions asked was also analyzed by Pearson correlation test. A p-value < 0.05 was considered statistically significant. The statistical analyses were performed using the Statistical Package for Social Science (SPSS, version 12) software package.

## Results

### Reliability

Cronbach’s α coefficient was used to assess reliability [9,10]. The Cronbach’s α values for the questionnaires of the 4 respondent groups were as follows: 0.696 for the students, 0.974 for the nurses, 0.914 for the teacher and 0.918 for the research assistant. The Cronbachs’ α values for the students’ scores would be 0.857 had the item “asking about chief complaints” been deleted. The item “asking about chief complaints” is of low reliability for the students’ assessing themselves in this study. The overall Cronbachs’ α value in this study was 0.867.

### Reasons for agreeing/disagreeing with video recording

A total of 60 family respondents of the 60 patients completed the questionnaires, 58 (96.7%) of them agreed with the video recording and is the source of data included for statistical analysis. Two reasons for reluctance were “personal privacy” issues and they “simply disagree” with video recording. Among the family respondents who agreed with the video recording, the following reasons were obtained: to contribute to medical education (41.8%), to get a more accurate diagnosis (13.9%), and to cooperate on the study (12.7%) (Figure 1).

### Satisfaction

The average satisfaction score of the 58 medical students was 85.1 points (references 60 points), indicating students’ performance was in the category between satisfied and very satisfied, which was derived from the following group scores: 88.9 points (family), 85.6 (research assistant), 84.0 (nurses), 82.2 (students) (Figure 2). All participants were most satisfied with the “teacher’s attitude” (92.9 points) and “teaching quality” (88.4 points). The family respondents were likewise most satisfied with the" “teacher’s attitude” (90.7 points), followed by the “clinic quality” (90.7 points); students were most satisfied with the “teacher’s attitude” (97.5 points), followed by “being open to questions” (92.5 points). In contrast, the family respondents were least satisfied with “being open to questions” (86.3 points), while the students were least satisfied with the “student’s attitude” (74.7 points) (Figure 2).

We discarded the students’ self-ratings as input in the t-test for differences between difficult and common cases. There was no significant difference in the overall satisfaction by the other 3 groups’ (family respondents, nurses, research assistant) evaluation between difficult cases and common cases (82.4 ± 13.8 vs. 85.6 ± 13.3, t = 1.481, degrees of freedom (df) = 172, P = 0.070). Lower scores were obtained in the difficult cases than in the common cases with regard to “student’s attitude” (80.0 ± 14.8 vs. 87.2 ± 13.5, t = 3.289, df = 172, P = 0.001), and “being open to questions” (79.3 ± 15.2 vs. 86.8 ± 12.8, t = 3.451, df = 172, P = 0.001). The number of questions asked was about 10–15 in each case. There was no significant correlation in the overall satisfaction with the number of questions asked (r = 0.021, P = 0.898).

### Evaluation of outpatient skill in 6 domains

Outpatient skills were assessed in 6 domains. Overall, students obtained the highest score on “explaining” (82.6 points) and the lowest score on “giving recommendations” (73.6 points).

The teacher assessed the students to have the highest score on the “explaining” outpatient skill (83.8 points), while the lowest was on “giving recommendations” (71.8 points). In the self-assessment of students, they had the highest score on the “explaining” skill (80.2 points) and the lowest on “providing recommendations” (72.5 points) (Figure 3). The teacher’s and the research assistant’s scores were comparable to the 4 groups’ average score. The nurses’ score was the highest and the students’ self-assessed score was lower than the average score of the 4 groups (Table 2).

**Table 2:** The total scores of outpatient skill in 6 domains between the different respondents

	**Mean**	**SD**
Teacher	75.6	8.2
Students	73.9	6.2
Assistant	75.9	7.1
Nurse	81.5*	4.4
Average	76.7	5.8

SD: standard deviation, * P < 0.05 compared to the average scores.

### Subdivided outpatient skills evaluation by the different groups

In the subdivided outpatient teaching skills, the students performed best in “asking patients about their chief complaints” (90.5 points), while worst in “asking about school/life burden” (65.6 points). Teachers believed that the students were best in “consensus decision maker” (94.5 points), followed by “giving self-introduction” (90.0 points). In contrast, they believed that the students were worst in “asking about school/life burden” (52.3 points), as did the students (66.4 points), nurses (62.3 points), and the research assistant (74.0 points). The second worst skill of the students was “Get a consensus with patient in different views” (average 69.2 points) (Table 1).

## Discussion

The agreement and satisfaction rate was high in this pediatric outpatient teaching evaluation. Different respondents will give much different scores in the evaluation. We noticed that the four respondents assessments could measure out more accurately the strengths and weaknesses of the students outpatient skills than the single one respondent assessment.

Why were their differences in satisfaction and skill scores between assessor groups? Were senior doctors more critical and strict in their assessments [11-13]?. In our study, we found that students gave more strict scores to themselves than the rest of the groups. Did the nurses give more favorable assessments because they understand the trainees better and thus make allowances for weakness [11,13]?. This favorable bias could be found in the nurses’ scores in our study, but not in the research assistant and the teacher. Alternatively, were their assessments more reliable because they know the students better [11]?. We found that the teacher’s and the research assistant’s scores were comparable to that of the 4 groups’ average score in Table 2. The nurses’ mean score was much higher and the students’ mean self-evaluation score was much lower than the average score. We believe that MSF containing four respondents assessments used in this study can give a more accurate assessment to distinguish students’ strong and weak points [14,15].

The quality of the results may also be influenced by personal relationships, stakes and equivalence [7]. Students’ own scores tended to be the lowest in our study. This is common in our teaching environment, and is probably due to the named questionnaires. Physician-patient communication has frequently been judged to be inadequate and imperfect [16]. It is thus important that communication skills of physicians be assessed periodically with a confidential peer evaluation survey [7], as such surveys may provide unbiased and factual information from respondents. However, in our study, preserving the anonymity and confidentiality of students and patients was difficult to achieve. First, selecting patients who were willing to participate in the study was not easy; second, the students who participated all completed the peer assessment in one outpatient room. However, this may be improved in the future by using a one-way mirror room or by ensuring that students complete the questionnaires in separate rooms.

Published findings showed that the number of questions asked about a patient’s illness was inversely related to patient satisfaction [17]. However, this inverse relation was not obtained in our study. We found that students assigned to difficult cases scored lower on “student’s attitude” and “being open to questions.” Counseling about unhealthy or risky behaviors is an important communication skill that should be a vital part of health care visits [2]. Physicians’ attitudes towards the physician-patient relationship may contribute to the diagnostic value of the patient history [18]. To this end, we should further educate the students about the appropriate attitude and approach in answering questions of patients with diseases that are difficult to treat. This may be an important future direction of research on physician-patient communication.

A patient-centered medical interview is essential to create good interpersonal relationships and information exchange; it may also contribute to the diagnostic value of the patient history and facilitate informed decision making [18,19]. Medical educators should focus on teaching and reinforcing behaviors that are known to enhance favorable patient outcomes and satisfaction [1]. Patient health outcomes can be improved with good physician-patient communication [19]. As Aspergren noted, communication skills can be taught in courses but are easily forgotten if not maintained by practice [20]. We believe that this pediatric-patient communication project will be helpful for the development and promotion of clinical skills of medical students in an outpatient practice. One limitation of this study was that this was a single site study using a specifically designed instrument and therefore might not be generalised. Therefore, these findings need to be tested in a large-scale study.

## Conclusions

MSF with video-recorded is important in providing a more accurate evaluation of students’ communication competence and in identifying the areas of communication that require enhancement.

## Abbreviations

CGMH: Chang Gung Memorial Hospital; MSF: Multisource feedback.

## Competing interests

None of the authors have financial disclosures or conflicts of interest to declare.

## Authors’ contributions

M-MT and L-TH provided the idea and framework for the study and edited the manuscript. M-MT wrote the primary manuscript draft. Y-HH, K-ST, CJ Chen performed the data analysis, generated the figures. All authors read and approved the final manuscript.

## Authors’ information

M-MT, L-TH, Y-HH, K-ST are staff in Chang Gung Memorial Hospital. M-MT is a physician educator in Chang Gung Memorial Hospital.

## Pre-publication history

The pre-publication history for this paper can be accessed here:

http://www.biomedcentral.com/1472-6920/13/145/prepub
